# A New Purpose of Cancer

**Published:** 1900-08

**Authors:** 


### A NEW PURPOSE OF CANCER.

AND now comes Clayton Jones nursing an idea in the *Lancet's* incubator of thought. The public or that portion of the public which has the good fortune to be interested in scientific matters sufficiently to take note of medical literature in passing, has been given a sort of pre-natal glimpse of Clayton Jones's idea and verily it is bizarre enough to entitle Clayton Jones to a front seat.

In his mental scientific combings Clayton Jones—there is a something delightfully rhythmical about that name--found an idea which to him opened up and explained away the whole cancer question. There was first presented to him the fact that a great many women have cancer after reaching a certain advanced age. Now, that was a fair enough beginning and Clayton Jones—that rhythm is certainly delicious—seized on it with the avidity of the incorrigible scientist and set him to work to find out why. To reach this end he made deductions. Heaven forbid that a reader of the JOURNAL should accompany the Jones gentleman through the brain twistings of his deducing journey; let it suffice that he deduced and that he gave birth to his idea which is that "modern civilisation has done away with the usefulness of women past the menopause," and that the appearance of cancer in elderly women is "nature's first effort to remove those useless individuals."

Naturally, after reading such an unblushing exposé of nature one marvels what manner of person this Jones gentleman is, that he should hold up one of the dearest institutions of youth—the grandmother—as a useless bundle of decaying tissue which his friend nature is saddling with the most horrible of all afflictions for the purpose of removing.

There are very few men who will not look with pity upon the Jones gentleman who attacks a class of women that from time immemorial have been the great bulwark of comfort and the refuge of safety to the great army of boyhood and girlhood. The grandmother occupies a warm corner in the heart of the world, the dear old, white-capped lady, with the soothing voice, the gentle, ministering touch, self-sacrificing and generous, whose faded eyes still shine with the love of life, whose silvern hair tells of years of toil and trouble, and joy and sadness, and work and the agony of maternity and the sweet joys of motherhood; whose face is deep scarred with the wounds of time, whose daily life and whose every act is anticipatory of the sweetness and the restfulness of the long sleep which is to come.

But then, maybe, Clayton Jones—does the name sound musical now?—never had a grandmother; maybe he never had a mother; maybe he's like Topsy; maybe he just "growed." Let us hope so anyhow. And then let us forget Clayton Jones—the man with the unnatural idea—Clayton Jones the confidante and friend of nature.

---

The New Haven Medical Society is at work on a law which has been too long on the statute books of Connecticut, whereby a judge can compel a physician to testify regarding matters which in the majority of states would be considered as privileged communications between physician and patient. The physicians in general all over the Nutmeg State are giving their support to the movement for the repeal of the law. It should be repealed. Such laws are obnoxious and distasteful. Physicians should not be compelled to divulge the statements made to them by patients, who when they tell facts to a physician, do so knowing full well that they are considered confidential and that only by so doing can a proper diagnosis be made and a cure effected.

---

After learning that the plague was carried and disseminated by rats the foreign scientists have unearthed a new and dangerous method of transmission. At Tokyo it has been demonstrated that fish are very generally infected with the plague bacillus. This, if true, is a serious question which the foreigners have to face, for fish is largely an article of diet in the section of the country in which the experiments were made. Crabs, too, have latterly been shown to be infected.

THE acquittal of Howard C. Benham was practically a victory of Buffalo. The majority of the scientists who appeared for the defence as medico-legal experts were Buffalo physicians and their evidence and their findings showed to the jury that, legally, Howard C. Benham was not guilty of the murder of his wife for which he had once been sent to Auburn to await execution in the electric chair. In connection with the acquittal of Benham a curious and interesting question has arisen. At the time he was in the death chamber at Auburn, merely awaiting the coming of the day which had been set for his death, the insurance company in which he was a policy holder offered to pay the full face of the policy—$6,000—rather than carry on its books a satisfied claim in the case of a murderer who had been executed. The company did not, of course, anticipate a new trial and an acquittal. They paid the insurance and now Benham is free and has had the benefit of the money which ordinarily is only paid after proofs of death have been filed. It is not known what the company will do; but the question arises, can Benham be insured in the same company again, it having already paid his death claim?

---

THE Knights of Honor at their recent convention in Buffalo discussed and decided two very important subjects at their sessions. They considered christian science and suicide. Christian scientists are admitted to membership and suicides are not barred after five years. The vote of christian science was 64 to 13. Whether the Knights needed the money or whether Mother Eddy did one of her white mahatma acts from a distance will never be known; but an interesting phase of the question will be the action of a christian scientist who happens to be appointed on the sick and benefit committee.

---

THE Trenton, N.J., Board of Health has nailed the tuberculosis flag to the mast head and issued an order placing that disease in the same category with smallpox, diphtheria, scarlet fever and other contagious and infectious diseases. Patients must be isolated and disinfected and hospitals are not exempt from the provisions of the order. Physicians are required under penalty of fine and imprisonment to report each case within thirty days after diagnosis and to send a sample of the sputum with the report. Trenton is in New Jersey, a state which has stood the jibes of the United States for years, but it has suddenly stepped to the front as a city with a progressive and fearless board of health. Trenton will wipe out tuberculosis, or come very near it with such a rule enforced.

Governor-General Leonard Wood, M.D., of Cuba, recently paid a timely visit to this country. He reports the rural districts of Cuba to be in excellent condition as to health, and that even the cities are free from the usual sickness that has heretofore prevailed. General Wood visited Boston and quieted the apprehensions of the Cuban teachers at Harvard, who feared they would soon be compelled to abandon their studies and return home for the want of funds. This, he said, was not the fact, but they would be allowed to finish their work. Cuba appears to be undergoing civil and sanitary regeneration at a rapid rate. Let us hope that our government will not be tempted by mistaken anti-imperialistic political shouting to relinquish its guiding hand too soon. Under the beneficent rule of the present Governor-General, Cuba is more prosperous than ever before and it is always safe to continue such an administration.

